# Best of Both Worlds: Adsorptive Ultrafiltration Nanocellulose‐Hypercrosslinked Polymer Hybrid Membranes for Metal Ion Removal

**DOI:** 10.1002/smsc.202400182

**Published:** 2024-08-11

**Authors:** Florian Mayer, Paul Schweng, Simone Braeuer, Sebastian Hummer, Gunda Koellensperger, Andreas Mautner, Robert Woodward, Alexander Bismarck

**Affiliations:** ^1^ Institute of Materials Chemistry and Research Faculty of Chemistry University of Vienna Waehringer Straße 42 1090 Vienna Austria; ^2^ Institute of Analytical Chemistry Faculty of Chemistry University of Vienna Waehrigner Straße 38 1090 Vienna Austria; ^3^ Institute of Environmental Biotechnology IFA‐Tulln University of Natural Resources and Life Sciences Vienna Konrad‐Lorenz‐Straße 20 3430 Tulln an der Donau Austria; ^4^ Division of Materials Science Department of Engineering Sciences and Mathematics Luleå University of Technology 971 87 Luleå Sweden; ^5^ Department of Chemical Engineering Imperial College London South Kensington Campus London SW7 2AZ UK

**Keywords:** hybrid membranes, hypercrosslinked polymers, ion exchange, nanocellulose, ultrafiltration

## Abstract

Efficient water treatment ideally combines ion exchange for the removal of hardness elements and toxic trace metals as well as ultrafiltration for the removal of particulate matter. Although promising for adsorption, many high‐surface‐area polymer materials cannot be easily processed into freestanding membranes or packed bed columns, due to poor solution processability and high back pressures, respectively. The preparation of hybrid membranes comprising sulfonated hypercrosslinked polymers entrapped in nanocellulose papers is described. The hybrid membranes are effective for simultaneous ultrafiltration and ion exchange. Increasing the polymer loading of the hybrid membrane produces synergy by increasing the permeance of the membranes while enhancing the ion adsorption capacity to values exceeding those of bulk hypercrosslinked polymers. The maximum ion adsorption capacity for copper is determined to be ≈100 mg g^−1^ outperforming that of pure polymer (71 mg g^−1^) and commercially available ion exchange resins. Competitive adsorption is tested in samples containing water hardness elements and trace toxic metal ions showing high ion‐exchange capacities. Even when fully loaded with water hardness elements, Ba^2+^ and Sr^2+^ are still removed from solution.

## Graphical abstract

A way to formulate sulfonated hypercrosslinked polymers (SHCPs) into hybrid membranes, a previously difficult task, utilizing a cellulose nanofiber network as a carrier matrix, is reported. The membranes combine the heavy metal adsorption capacity of SHCPs with the ultrafiltration capability of nanopapers, while showing synergistic effects of improved adsorption capacity and water permeance.

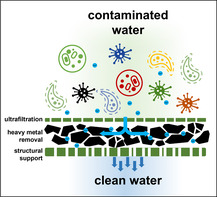

## 1 Introduction

Progressing industrialization and the accompanying increase of heavy metal and microplastic contaminated wastewaters significantly contribute to water scarcity by polluting ground and drinking water.^[^ ^1^, ^2^, ^3^ ^]^ Significant attention is being paid to the remediation of heavy metal ions which are toxic at high concentrations. Copper, for example, can cause damage to the immune system, the liver, the central nervous system, and has been identified to contribute to Alzheimer's and other neurodegenerative diseases.^[^ ^4^, ^5^ ^]^ Removal of heavy metal contaminants is performed in a variety of ways such as precipitation, coagulation, flocculation or flotation, electrochemical processes, membrane separation, and adsorption based methods.^[^ ^6^, ^7^, ^8^, ^9^, ^10^, ^11^ ^]^ Each method has advantages and disadvantages, especially with regard to their cost effectiveness, their environmental impact, and their ease of implementation.^[^ ^7^, ^11^ ^]^ Most water purification methods currently employed suffer from drawbacks such as high energy consumption, especially in the case of distillation technologies.^[^ ^12^ ^]^ For reverse osmosis and membrane distillation, a combination of significant water waste, membrane fouling, and/or low flux renders them cost ineffective for various scenarios.^[^ ^13^, ^14^ ^]^ Cost‐effective heavy metal removal methods are required and should preferably be based on sustainable materials and/or processes.^[^ ^15^, ^16^, ^17^ ^]^

Filtration is a size exclusion method widely used as a low‐cost approach for removing nonsoluble and even some soluble water pollutants. The main drawbacks of filtration are its inability to remove dissolved contaminants, for example, heavy metal ions, and its correlation between pore size and permeability. Adsorption is another widely used water treatment method in which contaminant materials are selectively extracted onto the surface of an adsorbent, such as ion exchange resins (IERs) or activated carbons.^[^ ^18^ ^]^ Although adsorption is effective for the removal of dissolved contaminants, drawbacks such as diffusion limitations and high backpressure have been reported, if implemented in packed beds.^[^ ^19^ ^]^ Hybrid solutions combining adsorption sites with (ultra)filtration, that is, filtration of particles <100 nm in diameter,^[^ ^20^ ^]^ offer effective (heavy metal) ion removal combined with high flux.^[^ ^21^, ^22^, ^23^ ^]^

Cellulose is a commonly used filter material, historically used in paper or cloth form for water or air purification.^[^ ^24^, ^25^, ^26^ ^]^ Cellulose nanofibers (CNF) are obtained by mechanical or chemical defibrillation of pulp fibers^[^ ^27^ ^]^ and are typically 5‐60 nm in diameter and several micrometers long.^[^ ^28^ ^]^ Papers made from CNF, called nanopapers (CNP), are considered next‐generation filter materials, as the dimensions of CNFs in CNPs enable ultrafiltration.^[^ ^25^, ^29^ ^]^ Surface modification of CNFs can enable heavy metal adsorption via the introduction of selective chemical moieties, which introduce desired surface charges.^[^ ^30^, ^31^ ^]^ However, such CNF functionalization's are often cost‐intensive and lead to increased filtration times required for paper formation. Hybridization of CNPs with state‐of‐the‐art adsorbent materials could offer a solution to this conundrum.

Potential candidate materials for hybridization are hypercrosslinked polymers (HCPs), amorphous, densely crosslinked porous organic polymers, produced using Friedel–Crafts chemistry.^[^ ^32^ ^]^ HCPs are a promising class of adsorbents for water purification, as their high surface areas provide abundant sorption sites and their hierarchical pore structures benefit mass transfer of adsorbates.^[^ ^33^ ^]^ We reported the synthesis of sulfonated HCPs (SHCPs) in a one‐pot approach, utilizing chlorosulfonic acid as a dual polymerization catalyst and sulfonating agent,^[^ ^34^ ^]^ resulting in SHCPs with higher sulfonation density and surface area as compared to conventional approaches.^[^ ^35^, ^36^ ^]^ SHCPs are useful for application as high‐capacity IERs.^[^ ^36^ ^]^ IERs have broad applications, including water softening, removal of heavy metals from wastewater, and separation of molecules by charge.^[^ ^37^ ^]^ However, porous organic polymers including HCPs have poor mechanical properties and are typically not solution processable, limiting their application as membrane materials. Nevertheless, studies have reported HCP‐based membranes accessible via hypercrosslinking of pre‐existing polymer membranes^[^ ^38^, ^39^ ^]^ and nanocomposite membranes for gas separation, comprising HCPs embedded in matrices of polydimethylsiloxane,^[^ ^40^ ^]^ poly[1‐(trimethylsilyl)‐1‐propyne],^[^ ^41^ ^]^ or even polymers of intrinsic microporosity.^[^ ^42^ ^]^

Here, we report hybrid membranes of SHCPs and CNFs for water treatment applications. We hypothesize that the use of CNF matrices will facilitate the homogeneous dispersion of embedded SHCP particles to produce membranes combining the ultrafiltration performance of CNP with the ion exchange capability of SHCPs. We manufacture multilayered CNF/SHCP hybrid structures using conventional filter paper carriers. By applying CNF top layers of varying thicknesses, membrane permeances are tailored to control the residence time of the permeate in the SHCP particle layer. The resulting hybrid membranes are tested for their ultrafiltration capabilities, heavy metal ion adsorption, and selectivity.

## 2 Experimental Section

### 2.1 Materials

4,4′‐Bis(chloromethyl)‐1,1′‐biphenyl (BCMBP, 95%), chlorosulfonic acid (99%), 1,2‐dichloroethane (DCE, ACS reagent, 99%), methanol (HPLC grade, ≥99.9%), ethylenediaminetetraacetic acid disodium salt dehydrate (EDTA), the indicator murexide, anhydrous calcium chloride, anhydrous copper chloride, and 10 nm‐diameter gold nanoparticles in citrate buffer were purchased from Sigma‐Aldrich. Magnesium chloride 6‐hydrate was purchased from BDH Laboratory supplies. Never‐dried elemental chlorine‐free bleached softwood pulp (*Picea abies* and *Pinus spp.*) was kindly provided by Stendal (Berlin, Germany) and comprised 81.3% cellulose, 12.6% hemicellulose, and 0.3% ash.^[^ ^43^ ^]^ Aqueous ammonia solution (25% p.a.) and hydrochloric acid (37%, p.a.) were purchased from Th.Geyer (Renningen, Germany). Nitric acid (≥69%, Rotipuran Supra) was obtained from Carl Roth. Mineral water (Longlife, Bad Radkersburg, Austria) was purchased in a local supermarket and ethanol (96% denatured with 1% petrolether) from Brenntag (Austria). Ultrapure water (18.2 MΩ cm, ELGA Water purification system, Purelab Ultra MK 2) was used for dilutions and calibration standards. Element standards were purchased from Labkings. If not stated otherwise, all materials were used as received

### 2.2 Synthesis of Sulfonated Hypercrosslinked Polymers

We synthesized SHCPs according to a modified procedure previously reported by us.^[^ ^34^ ^]^ Briefly, 4,4′‐bis(chloromethyl)‐1,1′‐biphenyl (10 mmol, 2.510 g) was dissolved in 1,2‐dichloroethane (DCE, 25 mL) at room temperature, before adding a solution of chlorosulfonic acid (40 mmol, 4.661 g) in DCE (5 mL). The reaction mixture was heated to 80 °C for 22 h. The resulting polymer was washed with methanol (≈100 mL) followed by a 24 h Soxhlet extraction using methanol followed by drying under vacuum at 80 °C overnight. We referred to the polymer as SHCP‐10 in accordance with our previous publication.^[^ ^34^ ^]^ The polymer was characterized as in the referenced publication. The full characterization procedure and data of SHCP‐10 are provided in the Supporting Information (Section S1).

### 2.3 Preparation of Cellulose Nanofibrils

Cellulose nanofibrils were prepared according to a procedure previously utilized by our group.^[^ ^44^ ^]^ In short, never‐dried cellulose pulp sheets were cut into 1 × 1 cm^2^ pieces and soaked in distilled water for at least 24 h before dispersing them using a mechanical blender (BL‐4473, Tristar) at 1500 W for 1 min. The cellulose suspension was subsequently passed ten times through a disc mill (MKCA6–23, Fuchs disc mill Granomat JP 150, Fuchs Maschinen AG) to refine the pulp to CNF. During the refinement process, the gap size of the disc mill was constantly reduced, and water was added as needed to ensure a consistency suitable for grinding. The resulting nanocellulose suspension was upconcentrated using gravity filtration to a dry mass content of 3.4 wt% before storage in an airtight container.

### 2.4 Hybrid Membrane Preparation

**Figure 1:**
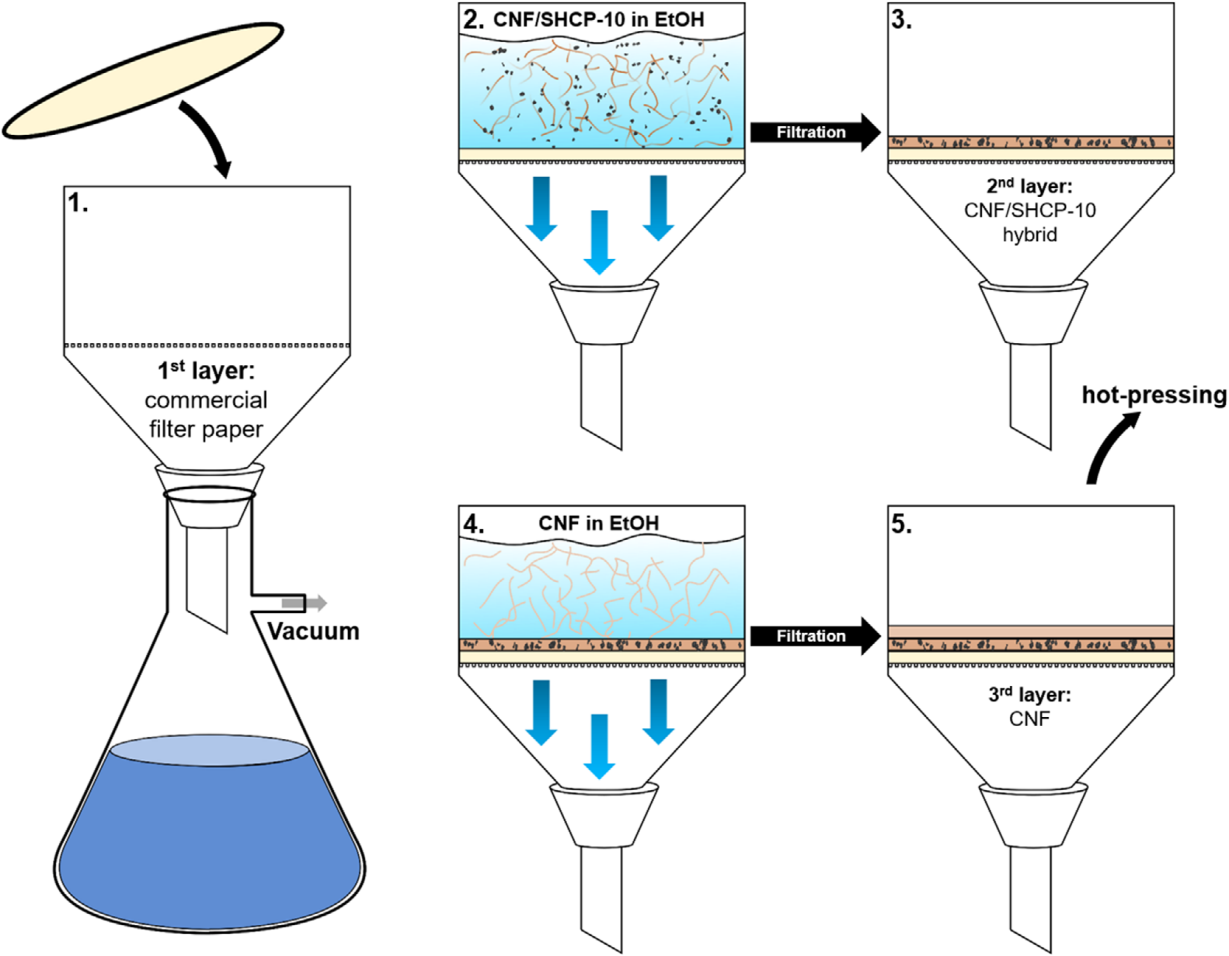
Schematic depiction of the hybrids manufacturing process utilizing a Büchner funnel in analogy to traditional papermaking techniques.

We developed a preparation technique for three‐layer hybrid membranes analogous to a traditional paper‐making procedure (**Figure** **1**). Hybrid membranes with varying amounts of CNF and SHCP were prepared in duplicate. Ethanol dispersions containing a 1:1 weight ratio of CNF and SHCP‐10 (0.2 wt% total) were filtered using a Büchner funnel (125 mm diameter) lined with a cellulose filter paper (5–13 μm pore size, VWR 413), which served as the bottom layer, to form a hybridized middle layer. A suspension of pure CNF in ethanol (0.2 wt%) was then filtered through the filter cake to form a top layer.

The resulting sandwich structure was placed between two steel plates, each lined with two layers of blotting paper (3MM Chr VWR) and pressed under 2 ton‐force for 10 s using a hydraulic press (type 25–12‐2H, Carver Inc.) at room temperature to remove excess ethanol. The hybrid membranes were then sandwiched between the same two steel plates lined with fresh blotting paper and release paper and pressed under 2 ton‐force at 120 °C for 15 min, to dry and consolidate the network. The composition of each hybrid, as well as their sample names, are shown in **Table** **1**.

**Table 1:** Sample code and layer grammages. Note that the middle layer grammage is the combined grammage of both constituents.

Sample Name	CNF/SHCP‐10 middle layer [g m^−1^]	Pure CNF top layer [g m^−1^]
SHCP/CNF 20‐5	20	5
SHCP/CNF 20‐10	20	10
SHCP/CNF 20‐25	20	25
SHCP/CNF 50‐5	50	5
SHCP/CNF 50‐10	50	10
SHCP/CNF 50‐25	50	25
SHCP/CNF 100‐5	100	5
SHCP/CNF 100‐10	100	10
SHCP/CNF 100‐25	100	25

### 2.5 SHCP‐10 Recovery from Spent Hybrid Membranes

An SHCP/CNF‐100‐10 hybrid membrane was submerged in 75 wt% sulfuric acid at room temperature for 1 h. The reaction was then quenched with 600 mL of ice cold water and centrifuged. After the supernatant was discarded, the polymer was resuspended in water, filtered, and washed until a neutral pH was reached.

### 2.6 Water Permeance Studies

The water permeance of hybrid membranes was determined using a dead‐end cell (HP4750, Sterlitech) with an active filtration area of 1460 mm^2^. Distilled water was passed through the membranes under a head pressure of 2 bar at room temperature. The permeate was collected in fractions and the water permeance P was calculated by measuring the volume *V* forced through the filter using

(1) $$P=\frac{V}{A\cdot t\cdot p}$$

where *A* is the active membrane area, *t* the collection time, and *p* the driving pressure. To ensure equilibrium was reached, the permeance was measured until a change of ≤2% occurred within 1 h.

### 2.7 Kinetic Studies of Cu^2+^ Adsorption on SHCP‐10

The adsorption kinetics of Cu^2+^ on SHCP‐10 was tested by suspending 100 mg of polymer in 25 mL of a 10 mmol L^−1^ CuCl_2_ solution. Samples of 100 μL were taken after 0.5, 1, 1.5, 2, 3, 5, 10, 60, and 1440 min and filtered through a syringe filter to remove any residual polymer. The fractions were analyzed using inductively coupled plasma‐mass spectrometry (ICP‐MS, Agilent 7800, Agilent Technologies) as this allowed for large numbers of samples to be measured efficiently. The instrument was operated in helium collision mode, to remove possible spectral interferences. Rhodium was added online via a T‐piece and used as an internal standard. Quantification was achieved via external calibration with pure element standards. The standard reference material 1643f (Trace elements in water, NIST) was used for quality control. All standards and samples contained 3% nitric acid for stabilization. Adsorption studies were carried out in triplicate at 30, 40, and 50 °C. The measured adsorption capacities at each point in time were fitted using pseudo‐first‐order and pseudo‐second‐order kinetic models (Equation (2) and (3), respectively):

(2) $$q_{t}=q_{e}\;\left(1-\;e^{-k_{1}t}\right)$$

(3)

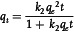

where *q*_*t*_ is the uptake of Cu^2+^ [mg g^−1^] at any given time *t* [min], *q*_*e*_ the equilibrium adsorption capacity [mg g^−1^], and *k*_1_ and *k*_2_ the first‐ and second‐order rate constant, respectively. The experimental data was fit with these models using an iterative nonlinear regression fit to reduce potential biases introduced by the uneven temporal spacing of sample collection. The fitting range was adjusted from *t* = 0 to the plateau region, indicating equilibrium (*t* = 1440 min for 30 °C, *t* = 60 min for 40 °C and *t* = 10 min for 50 °C), and the adsorption capacity at 24 h (1440 min) of all samples was reported as the experimental equilibrium adsorption capacity *q*_*e*,exp_. The initial sorption rate *h*_*0*_ was calculated using the pseudo‐second‐order model according to Equation (4).
(4)

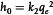

and the activation energy *E*_*a*_ of the reaction was calculated from the slope of the Arrhenius plot (ln(*k*_2_) vs. *T* ^−1^) according to Equation (5).

(5) $$\ln\left(k_{2}\right)=\frac{-E_{a}}{RT}+\ln\left(A\right)$$

### 2.8 Dynamic Heavy Metal Adsorption Tests of Hybrid Membranes

The dynamic metal adsorption capacity of CNF‐SHCP‐10 hybrid membranes was tested using a dead‐end flow cell with a filtration area of 1460 mm^2^. Circular discs were cut from the prepared hybrids and affixed in the cell, which was subsequently filled with 200 mL of CuCl_2_ solution (2.5 mmol L^−1^) and pressurized to achieve a flow rate of ≈1 drop s^−1^. A total of 35 fractions of 5 mL were collected and their concentrations determined by titration, following a procedure previously reported by our group.^[^ ^45^ ^]^ Briefly, 10 mL of supernatant was diluted in 50 mL water and the pH was adjusted to 10 by addition of an ammonium chloride/ammonia buffer. An aqueous solution of murexide indicator was added to the buffered supernatant until the solution turned yellow. This mixture was subsequently titrated against 0.1 mmol L^−1^ EDTA solution. The solution turned from yellow to red (equilibrium point) to a bright magenta. From the concentration of the supernatant before and after the adsorption experiment, the Cu^2+^ adsorption capacity *q* of SHCP‐10 was calculated according to Equation (6) and (7) by first calculating the mass of adsorbed Cu^2+^ $m_{\text{ads}}$ using the Cu^2+^ stock concentration $c_{S}$ and volume $V_{S}$, the titrant concentration $c_{\text{EDTA}}$ and volume $V_{\text{EDTA}}$, the molar mass of Cu $M_{\text{Cu}}$ (63.5 g mol^−1^), and the mass of SHCP‐10$m_{\text{pol}}$.

(6) $$m_{\text{ads}}=\left(\left(c_{S}\cdot V_{S}\right)-\left(c_{\text{EDTA}}\cdot V_{\text{EDTA}}\right)\right)\cdot M_{\text{Cu}}$$

(7) $$q=\frac{m_{\text{ads}}}{m_{\text{pol}}}$$

The concentration of the remaining supernatant in the dead‐end cell was also measured. The relative adsorption capacity q was determined from the amount of removed copper in all fractions analogous to the static adsorption tests and normalized to the SHCP‐10 content in the respective hybrid. The adsorption capacity per unit area $q_{A}$ of each hybrid membrane was calculated according to Equation (8) using the filtration area *A* (1460 mm^2^) of the dead‐end flow cell.

(8) $$q_{A}=\frac{m_{\text{ads}}}{A}$$

To evaluate membrane regeneration, 10 mL fractions of HCl solution (0.1 M, 120 mL total) were passed through sample SHCP/CNF 50‐10 to desorb Cu^2+^. The Cu^2+^ concentration in the washback fluid was analyzed as described above. Three adsorption cycles were performed.

### 2.9 Competitive Adsorption Studies

The capability of SHCP/CNF hybrids to remove common water hardness elements, such as Mg and Ca, and traces of toxic elements such as Sr and Ba were investigated, as well as selectivity. Commercial mineral water (LongLife, Bad Radkersburg, Austria) was diluted in a 1:1 ratio with ultrapure water (170 mL total) and filtered through SHCP/CNF 100‐5 at a pressure suitable to reach a permeance of ≈1 drop s^−1^ (0.5–0.8 bar). The eluent was collected in 5–10 mL fractions and analyzed using ICP‐MS as this method allowed for the simultaneous analysis of multiple elements. To test the ability of the SHCP/CNF hybrids to remove copper in the presence of the common water hardness elements Mg and Ca, a solution containing 1 mmol L^−1^ of Mg^2+^, Ca^2+^, and Cu^2+^ was prepared by diluting 101.7 mg of MgCl_2_·6H_2_O, 55.5 mg of CaCl_2_, and 67.2 mg of anhydrous CuCl_2_ in 500 mL of deionized water. This solution was treated in the same manner as the previously described mineral water and analyzed via ICP‐MS.

### 2.10 Particle Rejection Tests

To test the ultrafiltration capability of the manufactured membranes, 10 mL of a Au nanoparticle suspension (10 nm, 6 × 10^11^ particles per mL) were pushed through a 25 mm‐diameter SHCP/CNF‐20‐5 membrane at 2 bar of pressure using a dead‐end flow cell. The concentration of the eluent was determined by UV–vis spectroscopy (Agilent 8543 UV–vis Spectroscopy System, Agilent Technologies) using the absorption peak at 524 nm.

## 3 Results and Discussion

### 3.1 Sulfonated Hypercrosslinked Polymer Characterization

The full chemical and physical characterization of SHCP‐10 is provided in ESI Section S1. The chemical composition and physical properties agree with our previous findings.^[^ ^46^ ^]^

### 3.2 Copper Adsorption Kinetics on SHCP‐10

**Figure 2:**
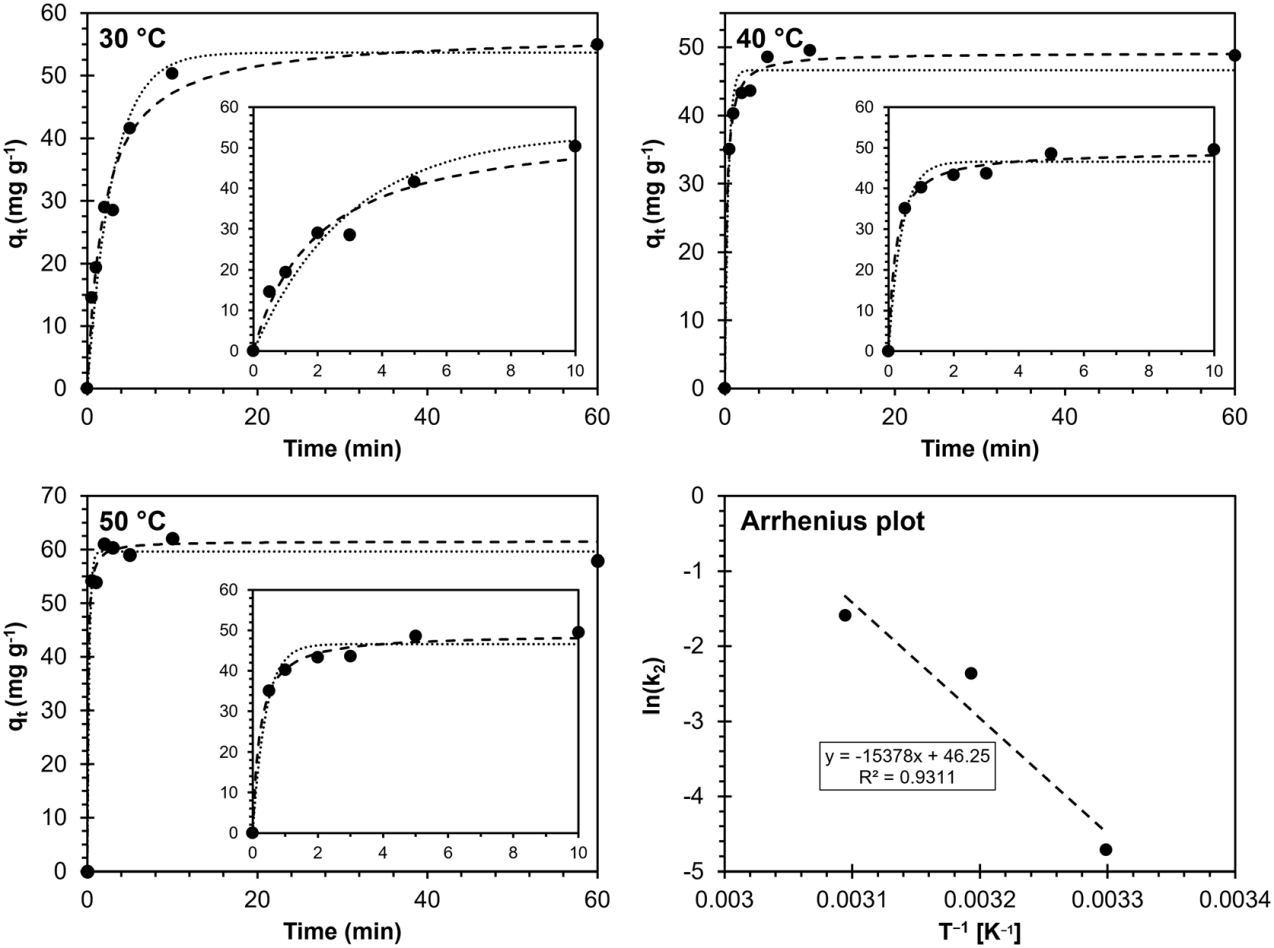
Adsorption studies at different temperatures T. The experimental data is depicted as solid dots, the pseudo‐first‐order and the pseudo‐second‐order model are represented by dotted and dashed lines, respectively. The bottom‐right graph presents the Arrhenius plot including the fitting function.

The results of the adsorption kinetic studies (**Table** **2** and **Figure** **2**) suggest that the pseudo‐second‐order model gives an accurate representation of the uptake mechanism of Cu^2+^ on SHCP‐10, in agreement with James et al.^[^ ^36^ ^]^ who found similar results for the adsorption of Sr and Cs. Analyzing the temperature dependency of *k*_2_ via an Arrhenius plot yielded an activation energy of 128 kJ mol^−1^ which, along with the pseudo‐second‐order nature of the uptake curves, suggests that the process is chemisorption based.^[^ ^47^ ^]^ The adsorbate interacts with specific binding sites—in this case the sulfonate groups—on the adsorbent's surface.

**Table 2:** Parameters obtained by fitting the experimental data of the adsorption of Cu^2+^ on SHCP‐10 to pseudo‐first‐ and second‐order models.

		Pseudo‐first‐order‐kinetic model			Pseudo‐second‐order‐kinetic model			
T [°C]	*q*_e,exp_ [mg g^−1^]	*q*_e,cal_ [mg g^−1^]	*k*_1_ [min^−1^]	R^2^	*q*_e,cal_ [mg g^−1^]	*k*_2_ [g mg^−1^ min^−1^]	*h*_0_ [mg g^−1^ min^−1^]	*R* ^2^
30	55 ± 5	54	0.34	0.9459	57	0.0090	29	0.9724
40	55 ± 4	47	2.5	0.8845	49	0.094	230	0.9720
50	59 ± 9	60	4.4	0.9276	62	0.20	770	0.9634

The experimental and fitted *q*_*e*_ values put SHCP‐10 in the same order of magnitude as a commercial IER AmberSep M4195 UPS (50 mg g^−1^) (Product data sheet AmberSepTM M4195 and AmberSepTM M4195 UPS Chelating Resins. Link in: https://www.dupont.com/content/dam/dupont/amer/us/en/water‐solutions/public/documents/en/IER‐AmberSep‐M4195‐and‐M4195‐UPS‐PDS‐45‐D00810‐en.pdf accessed on 18.01.2024) or adsorptive ultrafiltration membranes derived from polyvinyltetrazole‐co‐polyacrylonitrile (45 mg g^−1^)^[^ ^21^ ^]^ and significantly outperform phosphorylated nanocellulose fibers (20 mg g^−1^).^[^ ^48^ ^]^ The molar adsorption capacity of copper for SHCP‐10 is comparable to those for other SHCPs reported elsewhere when adsorbing Sr^2+^ and Cs^1+^ ions and considering the ionic charge.^[^ ^36^ ^]^

### 3.3 Manufacture of Three‐Layered SHCP/CNF Hybrid Membranes

The membrane preparation procedure was heuristically optimized over several preliminary tests, and issues, such as membrane cracking, low mechanical robustness, poor filtration performance, and heterogeneity of the polymer dispersion (Figure S2‐1, Supporting Information) had to be overcome. Eventually, ethanol was chosen as suspension medium as it produced CNF membranes with high porosity and flux^[^ ^49^ ^]^ and resulted in a uniform SHCP‐10 dispersion, which after filtration produced a homogenous distribution of SHCP‐10 throughout the adsorption layer.

**Figure 3:**
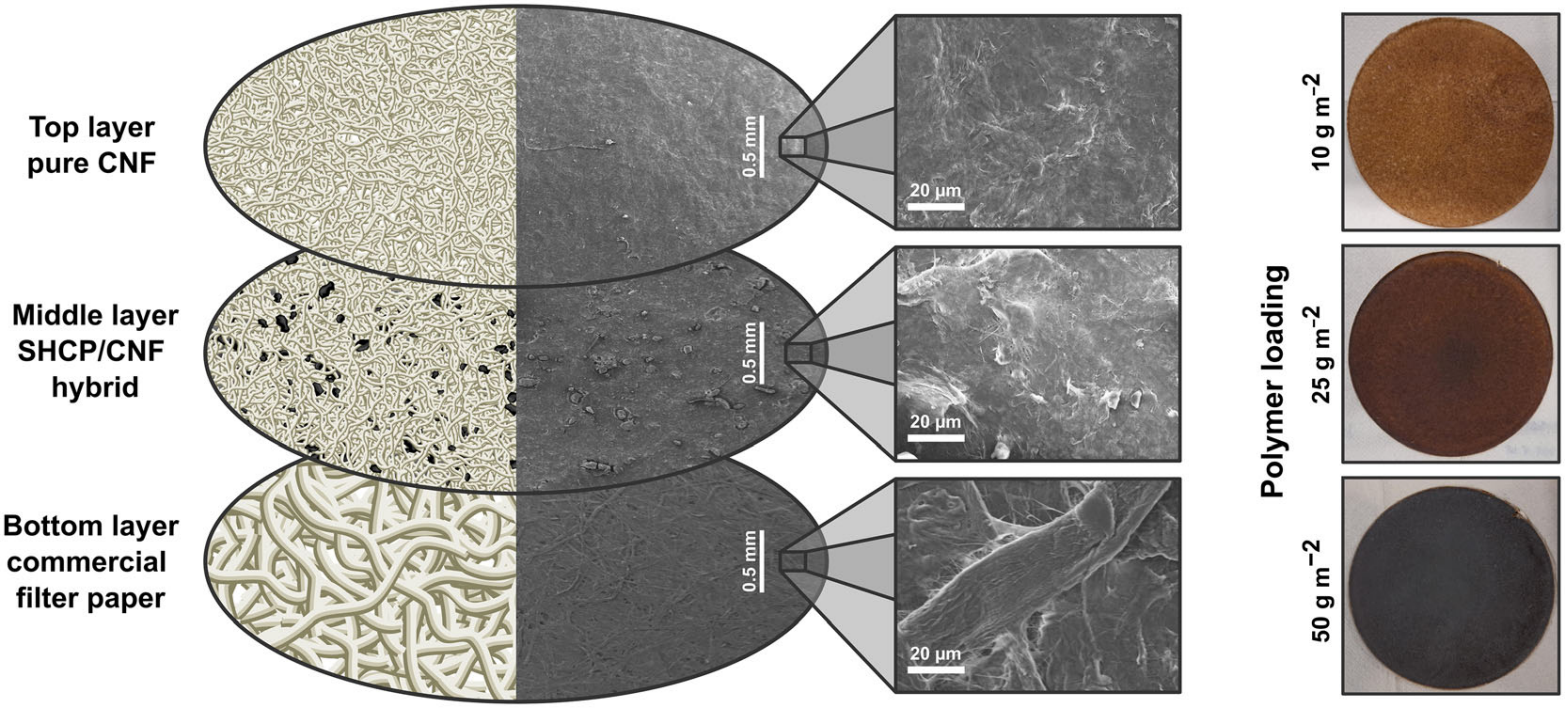
Composition of three‐layer SHCP/CNF hybrid membranes with accompanying scanning electron micrographs (left) and photographs of wet hybrid membrane filter cake precursors (membrane diameters are 125 mm) with varying middle layer polymer loading (right).

The general structure and scanning electron microscopy (SEM) images of an SHCP/CNF hybrid membrane are depicted in **Figure** **3**, showing a uniform, tight network of CNFs forming the top layer, small SHCP‐10 particles embedded within a CNF matrix for the middle layer, and large fibers constituting the commercial filter paper. An increasing middle layer grammage of the hybrids led to a darker color of the resulting filter cake (Figure 3), as a thicker layer of the brownish‐black SHCP‐10 was deposited.

### 3.4 Water Permeance of SHCP/CNF Hybrid Membranes

**Figure 4:**
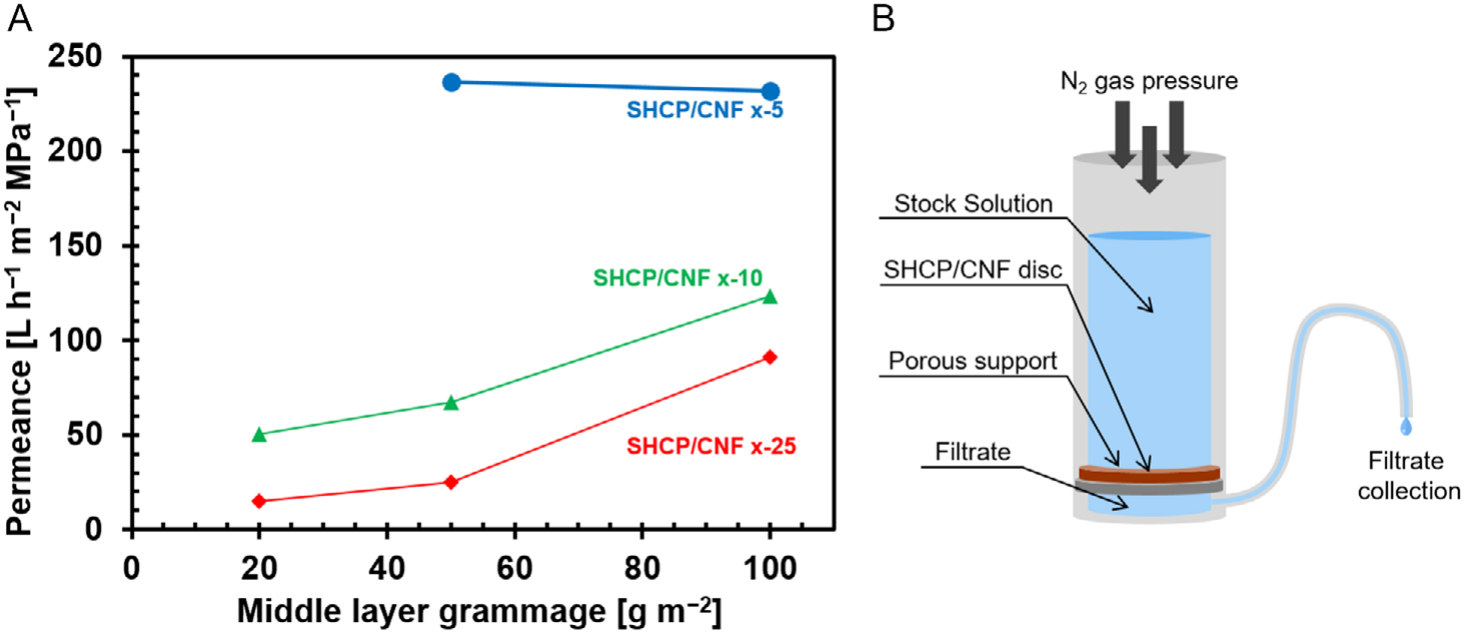
A) Equilibrium permeance of SHCP/CNF hybrid membranes as a function of middle and top layer grammage and B) a schematic depiction of the dead‐end cell test setup. Note: the errors are too small to be shown. Lines are provided to guide the eye and do not represent additional data points.

The equilibrium water permeance of the manufactured hybrid membranes is shown in **Figure** **4**A and was tested using a dead‐end flow cell (Figure 4B). The permeance of a typical measurement is depicted in Figure S3‐1 (Supporting Information), showing the gradual decrease of the permeance with filtration time until a stable plateau was reached. The drop in permeance was caused by compaction of the network under pressure, reducing the thickness and thus pore volume of the membrane. Membrane compaction led to a reduction in the amount of water able to pass the membrane per unit time.^[^ ^29^, ^50^ ^]^ Hybrid membranes with higher grammage reached equilibrium permeance faster, with SHCP/CNF 100‐25 equilibrating almost immediately. Reducing the thickness of the top layer increased the permeance of the hybrid membranes, indicating that the permeance is primarily controlled by the top layer thickness.^[^ ^29^, ^50^ ^]^

Thicker middle layers, that is, higher SHCP‐10 loading within the hybrids, led to an increased permeance for samples with 10 and 25 gsm top layers (SHCP/CNF x‐25 and SHCP/CNF x‐10, respectively), likely due to the incorporation of SHCP‐10 particles into the CNF matrix increasing pore volume and aiding channel formation. Hybrid membranes with 5 gsm top layers did not follow this trend as they already exhibited an overall much higher permeance. The water permeance of SHCP/CNF 20‐5 could not be tested as these hybrids were prone to pinhole formation due to the low cellulose content in both the middle and top layers.

To monitor possible SHCP‐10 leaching, the sulfur content of eluent fractions was determined using ICP‐MS and compared to that of the used DI water (Figure S3‐2, Supporting Information). The eluent contained no elevated sulfur content except for a marginal increase in the first fraction. The data suggests no continuous release of SHCP‐10 during use.

### 3.5 Copper Adsorption Capacity and Regeneration of SHCP/CNF Hybrid Membranes

**Figure 5:**
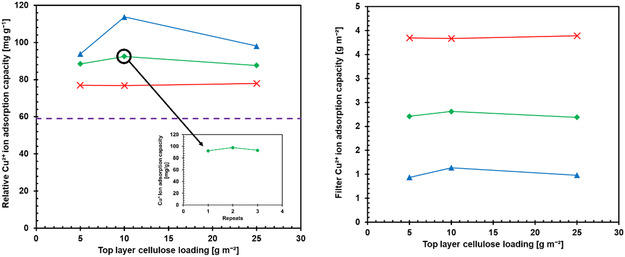
A) Relative Cu^2+^ adsorption capacity per gram of SHCP‐10 as a function of middle and top layer grammages of SHCP/CNF hybrid membranes and its performance after two regeneration cycles (inset). The purple dashed line corresponds to the highest batch sorption capacity of SHCP‐10. B) The absolute copper adsorption capacity per m^2^ of hybrid membrane. Note: Lines are provided as a guide to the eye and do not represent additional data points.

We determined the influence of the middle and top layer grammages of the hybrid membranes on Cu^2+^ adsorption capacity by dynamic adsorption (**Figure** **5**). Higher SHCP‐10 loading led to a slight decrease in the relative adsorption capacity per gram of SHCP‐10 (Figure 5A) yet increased the absolute Cu^2+^ adsorption capacity per membrane area (Figure 5B). The reduced relative capacity was attributed to the increased concentration of inaccessible SHCP‐10 particles or the formation of agglomerates. The relative adsorption capacity of SHCP‐10 in all hybrid membranes exceeded that of bulk SHCP‐10 in static adsorption tests, which was attributed to enhanced surface availability and the continuous replacement of depleted Cu^2+^ solution.

We regenerated SHCP/CNF 50‐10 by flushing with 120 mL of 0.1 M HCl solution (Figure 5A inset). SHCP/CNF 50‐10 was chosen as it was the median for both the investigated top and middle layer loading. We found that the relative Cu^2+^ adsorption capacity of the reused membrane did not decrease, demonstrating recyclability. Titration of the eluent fractions during regeneration showed that most of the copper was desorbed by the first 20–40 mL of HCl (Figure S4‐1, Supporting Information), demonstrating rapid regeneration and reusability of the hybrid membranes.

A comparison of the copper ion adsorption capacities of SHCP‐10 and SHCP/CNF hybrids with other materials for which regeneration is also reported is shown in Table S4‐1, Supporting Information. SHCP‐10 and its hybrids show an above average maximum Cu^2+^ adsorption capacity, ranging from 55 mg g^−1^ to 113.9 mg g^−1^. While SHCP/CNF hybrids do not possess the outstanding uptake capacities of, for example, glycine‐based magnetic nanoparticles^[^ ^51^ ^]^ or oxidized multiwalled carbon nanotubes,^[^ ^52^ ^]^ the ease of manufacture and ability to regenerate them without loss of material or function sets SHCP/CNF hybrids apart from a wide range of adsorbents.

### 3.6 Competitive Adsorption of Metal Ions on SHCP/CNF Hybrids

**Figure 6:**
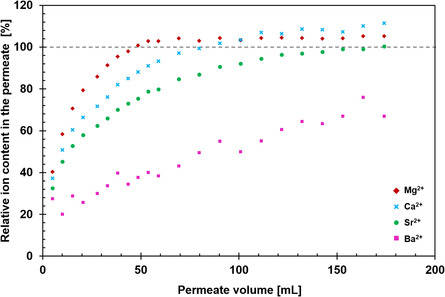
Relative breakthrough curves of various ions found in commercial mineral water upon treatment with hybrid membrane SHCP/CNF 100‐5. Typical relative standard deviations of the measurement are 2–3% and are not shown in the graph for legibility. The absolute concentrations of the relevant metal ions in the stock solution before passing through SHCP/CNF 100–5 were: Mg^2+^ = 82 mg L^−1^, Ca^2+^ = 109 mg L^−1^, Sr^2+^ = 3.2 mg L^−1^, and Ba^2+^ = 0.42 mg L^−1^.

We performed a competitive adsorption study on commercially available mineral water using SHCP/CNF 100‐5, as this hybrid combined high permeance with high absorption capacity per unit area. Commercial mineral water was chosen as a complex feed containing the most important water hardness elements, Ca and Mg, as well as traces of other problematic alkaline Earth metals including Sr and Ba. The radioactive isotopes of strontium are used as radiopharmaceuticals (^89^Sr)^[^ ^53^ ^]^ and are present in nuclear waste and fallout (^90^Sr).^[^ ^54^ ^]^ Barium is commonly used as a weighing agent in drilling fluids of oil and gas wells^[^ ^55^ ^]^ and is considered neurotoxic. The relative breakthrough curves (i.e., the percentage of each ion compared to its feed concentration) are presented in **Figure** **6**.

The relative breakthrough curves (Figure 6) show the capability of SHCP/CNF hybrids to exchange ions effectively and, thus, remove, for example, alkaline Earth metals from solutions. The tested concentrations simulate naturally occurring conditions, in which Mg and Ca are present in significant excess of Ba^2+^, present in trace amounts. In the first fraction, 60–70% of all investigated ions were removed. In subsequent fractions, the removal of Ca^2+^ and Mg^2+^ ions decreased due to saturation of sulfonate sites. In contrast, the removal of Ba^2+^ and Sr^2+^ from the stream continued for the entirety of the experiment. The removal efficiency of each alkaline Earth metal appeared to follow that of the solubility of the sulfate salts of the individual ions: MgSO_4_ (pK_sp_ = −0.981) > CaSO_4_ (pK_sp_ = 3.64) > SrSO_4_ (pK_sp_ = 6.46) > BaSO_4_ (pK_sp_ = 9.97).^[^ ^55^ ^]^ The state of chemical equilibrium of Ca^2+^ and Mg^2+^ ions with SHCP‐10's sulfate moieties implies that there are always a number of free sulfonate sites, which could also be repopulated with the much less soluble Sr^2+^ and Ba^2+^ counterions, leading to an increased relative concentration of Mg and Ca in the permeate of later fractions.

**Figure 7:**
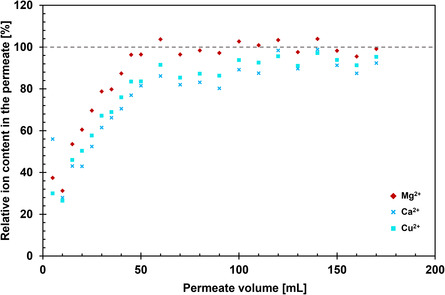
Relative breakthrough curves of an artificial wastewater sample containing Mg^2+^, Ca^2+^, and Cu^2+^ ions upon treatment with hybrid membrane SHCP/CNF 100‐5. Typical relative standard deviations of the measurement are 2–3% and are not shown in the graph for legibility. The absolute concentrations of the metal ions in the solution before passing through SHCP/CNF 100‐5 were 1 mmol L^−1^, corresponding to Ca^2+^ = 40 mg L^−1^, Mg^2+^ = 24 mg L^−1^, and Cu^2+^ = 63.5 mg L^−1^.

The ability of SHCP/CNF hybrids to adsorb Cu^2+^ ions in the presence of Mg^2+^ and Ca^2+^ was tested on an aqueous solution containing 1 mmol L^−1^ of each ion (**Figure** **7**). The solution was formulated to simulate water with copper‐contaminated wastewater with high‐to‐medium hardness, as can be found for example in the effluent wastewater of mirror‐making industries.^[^ ^48^ ^]^

The breakthrough curves of water containing Cu^2+^, Mg^2+^, and Ca^2+^ ions follow the same trend as those of commercial mineral water and show that the membranes are able to adsorb copper ions in the presence of common water hardness elements, albeit without any specificity toward copper. The marginal selectivity toward individual ions once more follows the solubility of the respective ions’ sulfate salts (MgSO_4_ (pK_sp_ = −0.981) > CuSO_4_ (pK_sp_ = 1.72) > CaSO_4_ (pK_sp_ = 4.31)).^[^ ^55^ ^]^

### 3.7 Ultrafiltration Ability of SHCP/CNF Hybrid Membranes

We assessed the ultrafiltration capability of our hybrid membranes via size exclusion of 10 nm Au nanoparticles. SHCP/CNF 20‐5 was chosen as the substrate as it was the thinnest sample produced in our study, and it is assumed that thicker samples (i.e., hybrids with higher top and/or middle layer grammage) will display at least equivalent or rather improved particle rejection. The UV–vis spectra (Figure S5‐1, Supporting Information) of the Au nanoparticle suspension feed and permeate indicated full rejection of the nanoparticles. The full rejection of 10 nm particles confirms that SHCP/CNF hybrid membranes are able to remove, for example, microplastics (smallest reported sizes of ≈1 μm)^[^ ^56^ ^]^ and/or even the smallest existing viruses, which are ≈20 nm in size.^[^ ^57^, ^58^ ^]^

During permeance, adsorption, and particle rejection testing, the samples mounted in dead‐end flow cells were subjected to hydraulic pressures of up to 2 bar, without failure occurring. The commercial filter paper used as support constitutes a major portion of the hybrid membranes; thus, its mechanical properties determine that of the hybrids. The filter paper used has a wet and dry burst pressure of 0.6 and 1.9 bar, respectively (VWR product catalogue “VWR for filtration” Link in: https://media.vwr.com/ecatalog/index.html?catalog=EU_AIO_Filtration_2015/EN&shop=uk accessed 10.04.2024), suitable for a myriad of common filtration and ion exchange applications.

### 3.8 Recovery of SHCP‐10 from SHCP/CNF Hybrids

The recovery of SHCP‐10 from a used hybrid membrane was investigated by controlled acid digestion of the surrounding CNF matrix. Full polymer characterization was performed and showed no significant change compared to as‐synthesized SHCP‐10 apart from a slight increase in carbon content and a miniscule decrease in surface area. These changes were due to the presence of undigested carbonization products from the degradation of cellulose during acid digestion (ESI Section 1).

## 4 Conclusion

We introduced HCP–cellulose nanofibril layered hybrid membranes to utilize the advantageous features of both materials. CNF constitutes the carrier material, lending structural stability to the otherwise powderous SHCP‐10 while functioning as a tight ultrafiltration network facilitating rejection of nanoparticles down to 10 nm. The incorporation of SHCP‐10 into the hybrid membrane increased water permeance. SHCP‐10 adsorbed high concentrations of Cu^2+^ from aqueous solutions in both static and dynamic experiments (up to 114 mg g^−1^). The most promising membrane, SHCP/CNF 100‐5, was used in the treatment of a commercial mineral water containing not only standard water hardness metal ions (Mg^2+^ and Ca^2+^) but also trace amounts of toxic heavy metals (Sr^2+^, and Ba^2+^). In addition to water softening, hybrid membranes also removed trace toxic Sr^2+^ and Ba^2+^ even after saturation of sulfonate groups in the presence of excess concentrations of Ca^2+^ and Mg^2+^.

## Conflict of Interest

The authors declare no conflict of interest.

## Author Contributions

**Florian Mayer** and **Robert Woodward** conceived the idea. **Florian Mayer** performed ultrafiltration and metal adsorption experiments. **Florian Mayer** and **Paul Schweng** prepared hybrid membranes, performed flux tests, and conducted recovery experiments. **Paul Schweng** prepared and characterized SHCP‐10. **Sebastian Hummer** conducted kinetics experiments. **Simone Braeuer** and **Gunda Koellensperger** analyzed metal ion content of solutions. **Alexander Bismarck** made **Florian Mayer** perform competitive adsorption measurements, statistics, and ensure reproducibility. **Alexander Bismarck**, **Andreas Mautner**, and **Robert Woodward** supervised this work. **Florian Mayer**, **Robert Woodward**, and **Alexander Bismarck** prepared the draft manuscript and all authors discussed, edited, and commented on the manuscript. **Alexander Bismarck** secured funding.

## Supporting information

Supplementary Material

## Data Availability

The data that support the findings of this study are available from the corresponding author upon reasonable request.
