# Supplementary material for: Best of Both Worlds: Adsorptive Ultrafiltration Nanocellulose‐Hypercrosslinked Polymer Hybrid Membranes for Metal Ion Removal
Source: Small Sci. 2024 Aug 11;4(10):2400182. doi: 10.1002/smsc.202400182 (PMC11935042; doi:10.1002/smsc.202400182)
Supplement: Supplementary file 1 — Supplementary Material [file SMSC-4-2400182-s001.pdf]

Supporting Information

Best of both worlds: Adsorptive ultrafiltration nanocellulose-hypercrosslinked polymer hybrid membranes for metal ion removal

Florian Mayer[a], Paul Schweng[a], Simone Braeuer[b], Gunda Koellensperger[b], Andreas Mautner[a,c], Robert Woodward[a], Alexander Bismarck[a,d,e]

a Institute of Materials Chemistry and Research, Faculty of Chemistry, University of Vienna, Waehringer Straße 42, 1090, Vienna, Austria.

b Institute of Analytical Chemistry, Faculty of Chemistry, University of Vienna, Waehrigner Straße 38, 1090, Vienna, Austria

c Institute of Environmental Biotechnology, IFA-Tulln, University of Natural Resources and Life Sciences, Vienna, Konrad-Lorenz-Straße 20, 3430, Tulln an der Donau, Austria

d Division of Materials Science, Department of Engineering Sciences and Mathematics, Luleå University of Technology, Luleå, 971 87, Sweden

e Department of Chemical Engineering, Imperial College London, South Kensington Campus, London SW7 2AZ, United Kingdom

SI 1. Sulfonated hypercrosslinked polymer characterisation

Finely ground samples were analysed using Fourier-transform infrared (FTIR) spectroscopy using a Bruker Tensor II FTIR spectrometer equipped with a Platinum ATR module (Bruker) and constantly flushed with dry air. Spectra were acquired in double-sided forward backward acquisition mode with a resolution of 4 cm−1 in the range of 400-4000 cm^{-1} and the obtained spectra were averaged over a total of 32 scans using Blackman–Harris 3-term apodisation function and a zero-filling factor of 4.

Thermogravimetric analyses were performed using a TA instruments Discovery TGA. Platinum sample pans were loaded with approximately 20 mg of sample and ramped at a rate of 10 °C·min^{-1} under either air or N_2 gas flow (100 mL·min^{-1}) from room temperature to 800 °C.

Elemental analysis was performed using a Eurovector EA 3000 CHNS-O Elemental Analyser. 0.75-3.0 mg of each sample was weighed into tin vials (4×6 mm) using a microbalance (Sartorius, ME 5 OCE). Samples were run in triplicate. The operating temperatures for the combustion and reduction were 1000 °C (1480 °C for O analysis) and 750 °C, respectively. He (99.999+) was used as carrier gas.

Porous properties of SHCP were determined at –196 °C using N_2 gas sorption (TriStar II, Micromeritics). Samples were degassed (FlowPrep 060, Micromeritics) for at least ten hours at 120 °C under constant N_2 flow. For surface area calculations the Brunauer-Emmett-Teller (BET) model was applied to the adsorption branch in the range of 0.05-0.2 P/P_0. The total pore volume was calculated from the amount of N2 adsorbed at P/P_0 = 0.97. For the determination of the micropore volume the t-plot method was applied to the adsorption branch in the range of 0.15-0.4 P/P_0.

The elemental composition of SHCP-10 was determined by X-ray photoelectron spectroscopy (Nexsa, Thermo Fischer) using Al K_α X-rays at 72 W using a spot size with 400 μm diameter. Survey spectra with resolutions of 1 eV, a pass energy of 200 eV and a dwell time of 10 ms were recorded and averaged over 50 scans. High resolution spectra for carbon (C1s 279-298 eV), oxygen (O1s 525-545 eV), nitrogen (N1s 392-410 eV), chlorine (Cl2p 190-210 eV) and sulfur (S2p 157-175 eV) were recorded with a resolution of 0.1 eV, a pass energy of 50 eV, a dwell time of 50 ms, and were averaged over 30 scans. The spectra were analysed using the software package Avantage (ver. 5.9931, Thermo Scientific, East Grinstead, UK) and the elemental composition calculated from the respective peak areas using the included ALTHERMO1 scaling factor database.

The size distribution of SHCP-10 particles was determined via LASER-diffraction testing using a HELOS KR (Sympatec GmbH, Clausthal-Zellerfed, Germany) fitted

with a RODOS dry dispersion unit and utilising the incorporated SUBMICRON Fourier-lens system with a focal length of 50 nm. The experiment was carried out in duplicate.

The successful synthesis of SHCP-10 was confirmed by ATR-FTIR (Fig. SI 1-1). Bands assigned to S=O and C-S stretching vibrations were found at 1140 cm^{-1} and 600 cm^{-1}, respectively.

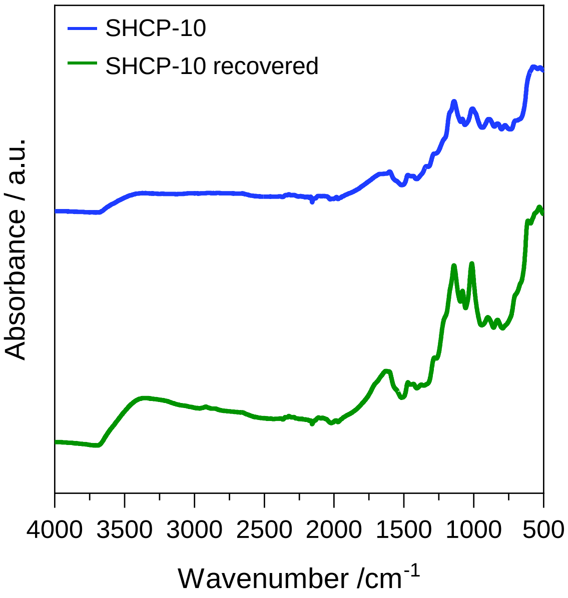

Figure SI 1-1. ATR-FTIR spectra of pristine and recovered SHCP-10

The elemental composition of SHCP-10 was determined via X-ray photoelectron spectroscopy (Table SI 1-1) and showed a sulfur content of 6.4 at.-%, in good agreement with our previous work. Elemental analysis (Table SI 1-2) gave sulfur values of ~3.8 at.-%, lower than that measured by XPS. The difference in sulfur content between the two methods is attributed to water adsorption due to the strongly hydrophilic nature of SHCP-10 prior to EA, [1] as well as the inability of XPS to consider

hydrogen. The actual sulfur content likely lies somewhere between the results of XPS and EA. [2] ,

Table SI 1-1 Atomic composition of SHCP 10 after synthesis and after reclaim via acid digestion as determined by XPS

	C (at.%)	O (at.%)	S (at.%)
**SHCP-10**	70.6	20.8	6.4
**SHCP-10_recycled**	72.2	19.9	6.0

Table SI 1-2. Atomic composition of SHCP 10 after synthesis and after reclaim via acid digestion as determined by elemental analysis

	C (wt.%)	H (wt.%)	N (wt.%)	O (wt.%)	S (wt.%)
**SHCP-10**	42.07 ± 0.39	4.73 ± 0.13	0.05 ± 0.00	36.70 ± 0.16	13.41 ± 0.09
**SHCP-10 recovered**	50.65 ± 0.33	4.32 ± 0.09	0.20 ± 0.01	31.58 ± 1.01	10.83 ± 0.14

The N_2 gas sorption isotherms (Figure SI 1-2) showed characteristics of both type I and type IV(a), as described by IUPAC. [3] A steep slope in the low-pressure region ($P/P_0 < 0.1$) confirmed microporosity and the hysteresis stemming from capillary condensation in the high relative pressure region ($P/P_0 > 0.9$), is indicative of mesopores.

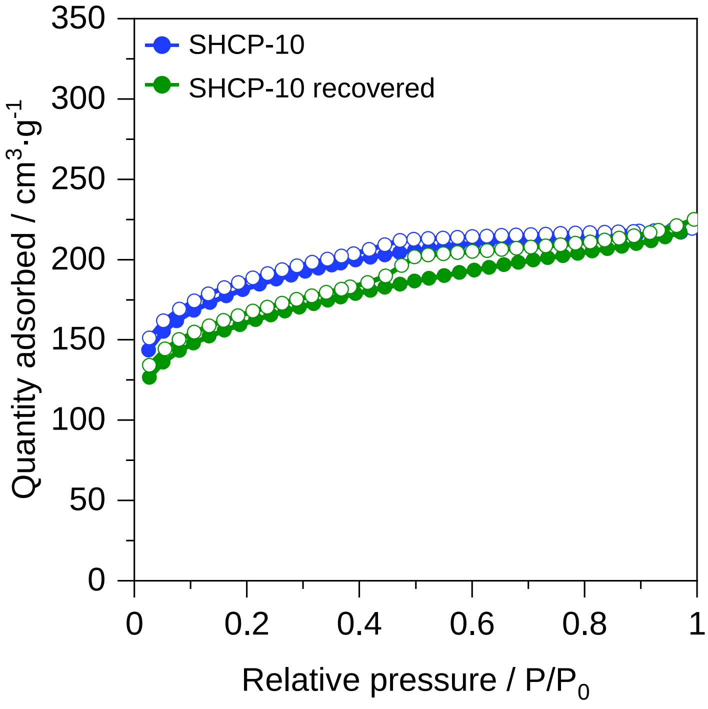

Figure SI 1-2. BET plots of virgin and recovered SHCP-10

The BET specific surface area was determined to be 697 ± 35 $m^2 \cdot g^{-1}$ $m^2 \cdot g^{-1}$ and the micropore and total pore volume was 0.17 ± 0.01 $cm^3 \cdot g^{-1}$ and 0.38 ± 0.04 $cm^3 \cdot g^{-1}$, respectively.

Thermogravimetric analysis in air atmosphere (Figure SI 1-3) showed an initial weight loss of 23 wt.-% up to 120°C, which was attributed to the desorption of water, after which the weight was stable until ~200 °C, at which point the decomposition of the sulfonategroups takes place.

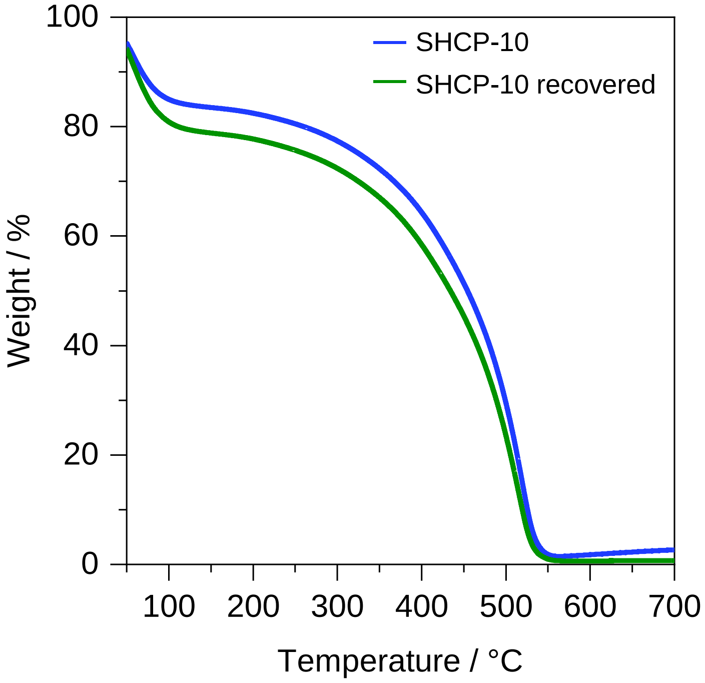

Figure SI 1-3. TGA graphs in air of virgin and recovered SHCP-10

We explored the possibility to recover SHCP at the end of a membranes use cycle via controlled digestion of the surrounding CNF in 75 wt.-% sulfuric acid at room temperature. Post-treatment, the N_2 sorption isotherms (Figure SI 1-2) of recovered SHCP indicate a slight decrease in sorption at the meso/macropore region, resulting in a decrease in BET specific surface area from 697 ± 35 $m^2 \cdot g^{-1}$ to 504 ± 60 $m^2 \cdot g^{-1}$, paired with a decrease in total and micro pore volume . Upon re-analysis, the network displayed some reduction in sulfonic acid group density, apparent through a decrease in sulfur content from 3.84 ± 0.03 to 3.12 ± 0.04 at.-%, according to EA and 6.4 to 6.0 at.-%, as determined by XPS. The decrease in sulfur content was accompanied by an increase in carbon content, which we hypothesise is due to some residual carbon from CNF acid degradation. Additional physical and chemical characterisations, including FTIR (Figure SI 1-1) and TGA (Figure SI 1-3) revealed no measurable change.

The particle size distribution of hand-ground SHCP-10 (Figure SI 1-4 left) shows that the majority of the particles are in the 20 to 40 µm range. It should be noted that small quantities of larger particles (120 µm and above) were observed in SEM (Figure SI 1-4 right), which are outside of the measuring range for LASER-diffraction experiments.

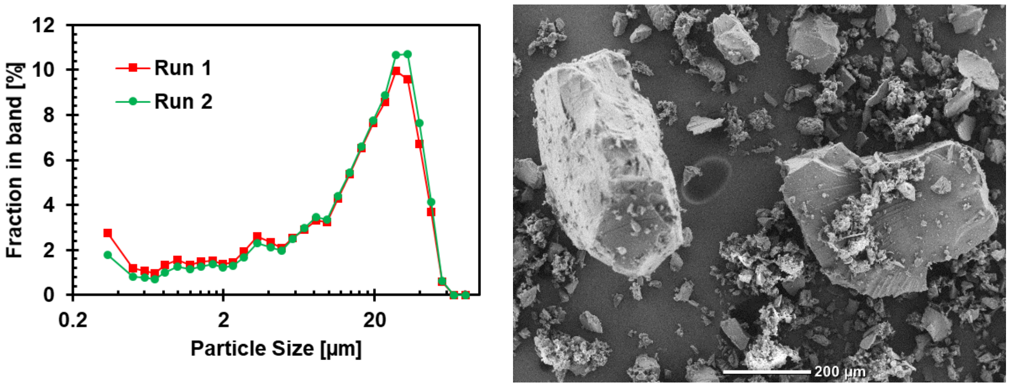

Figure SI 1-4: Particle size distribution of SHCP-10 used for the manufacturing of hybrids (left) and an SEM image showing the larger particles found.

SI 2. Results of preliminary hybrid manufacturing tests

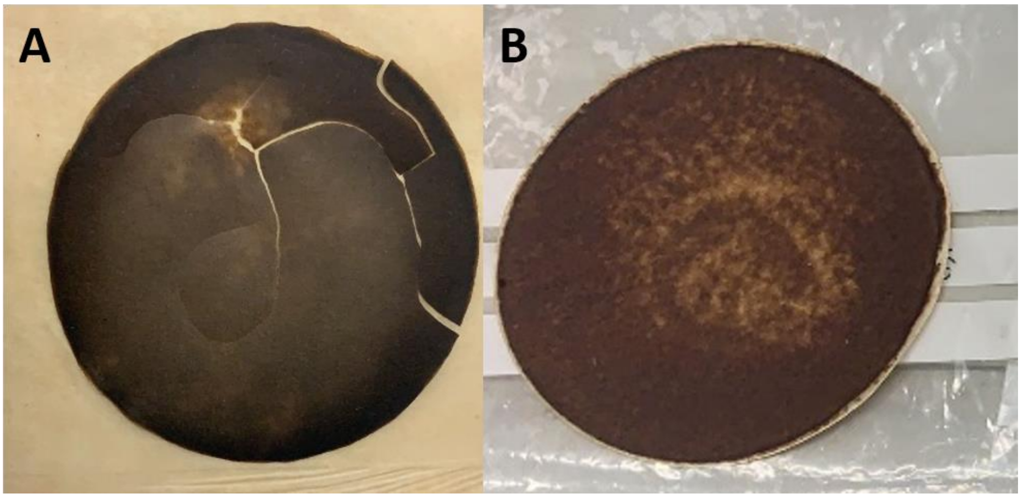

Figure SI 2-1: Photographs of defective SHCP/CNF hybrids. Increasing the polymer content of the middle layer above 50 wt.-% led to cracking during hot pressing (A) and using water as suspension medium led to uneven deposition and agglomeration of SHCP-10.

SI 3. Water permeance measurements

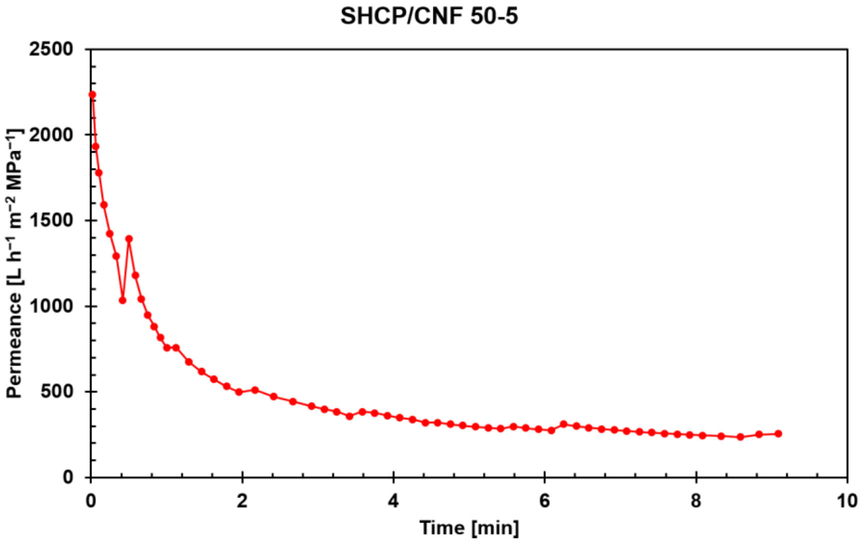

Figure SI 3-1: Representative permeance over time curve as recorded during water permeance tests. The sudden increase in permeance for some points is caused by the water changes necessary to refill the setup which in turn depressurise the system,

allowing for the compressed hybrid paper to relax to a certain extent, increasing the flow rate briefly.

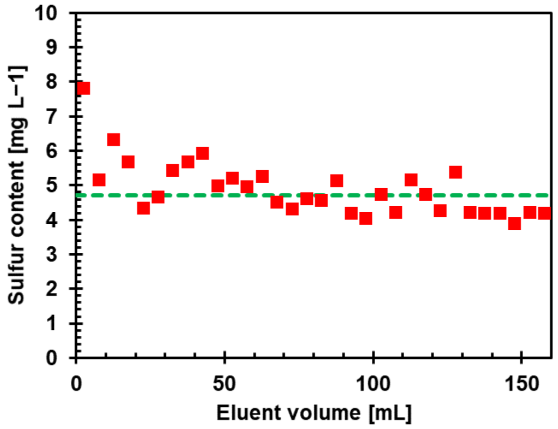

Figure SI 3-2: Sulfur content of the eluent fraction of pure water after passing through a hybrid (red dots) vs the sulfur content of the used stock solution (green dashed line). Note: these values were obtained via single measurements.

SI 4. Cu^{2+} desorption during regeneration

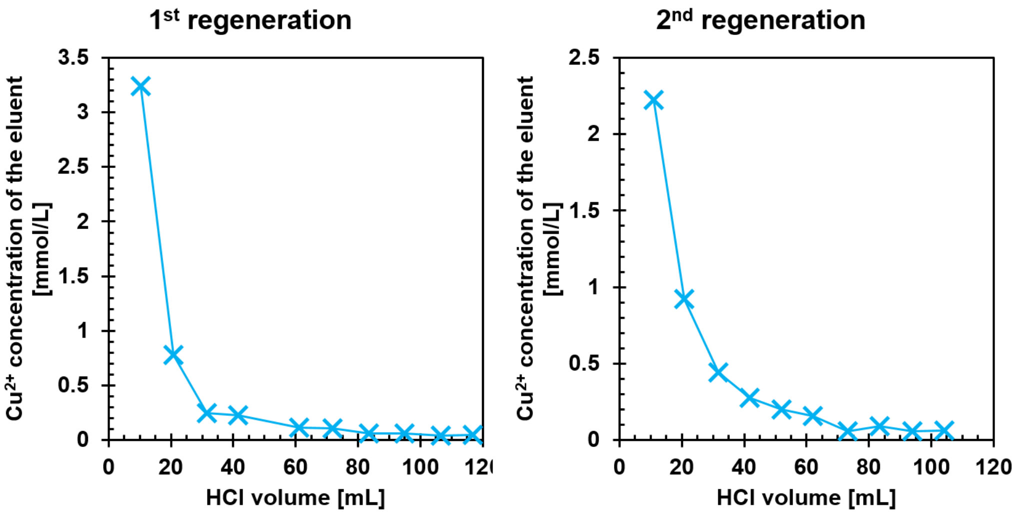

Figure SI 4-1 Concentrations of the eluent fractions during Regeneration of SHCP/CNF 50-10

Table SI 4-1 Comparison of copper adsorption capacity and regeneration performance of a variety of materials with SHCP-10 and SHCP/CNF hybrids.

**Adsorbent**	q_{max} **(mg/g)**	**Regeneration**		**Ref.**
		**Number of Cycles**	**%** q_e **retained**	
Glycine functionalized magnetic nanoparticles	625	4	89	[4]
Oxidised multiwall carbon nanotubes	416.5	6	87	[5]
Carboxylated graphene oxide	357.1	5	96	[6]
Schiff base ligand nanocomposites	173.6	7	92	[7]
Graphene nanosheet/MnO_2	103	4	–	[8]
2D $Ti_3C_2T_x$ MXene Nanosheets	78.45	3	30	[9]
Chitosan/Ag nanoparticle/Cu nanoparticle/carbon nanotube composites	70.4	5	approx. 60	[10]
Pectin functionalised Fe_3O_4 nanoparticles	48.99	5	58.66	[11]
Bovine manure compost	29.9	3	>99	[12]
Poly(methacrylamide-co-acrylic acid)/montmorillonite nanocomposites	29	5	90	[13]
diethylenetriamine functionalized SiO_2/Fe_3O_4 nanoparticles	13.46	3	75	[14]
SHCP-10	55-59			this study
SHCP-10 in SHCP/CNF membranes	77.9-113.7	3	>99	this study

SI 5. Au nanoparticle rejection tests

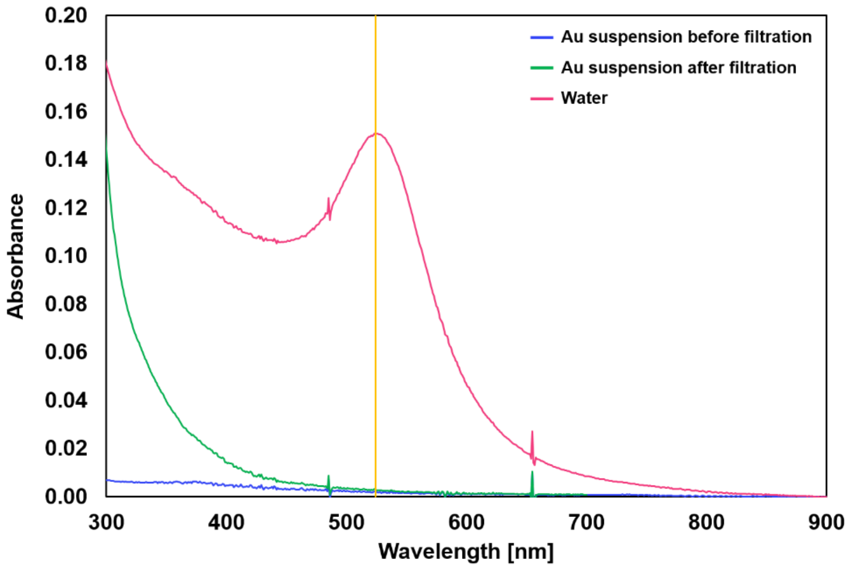

Figure SI 5-1 UV-Vis spectra comparing Au NP stock solution before (red) and after (green) passing through SHCP/CNF 20-5. The spectrum of water is included as reference. The yellow line denotes the peak maximum at 524 nm.

The absorbance peak at 524 nm, attributed to the Au nanoparticles, was reduced to the equivalent of pure water after filtration. Only the adsorption peak of the citric acid stabilizer exhibiting its maximum below the tested wavelength range remained.

References

[1] Schweng, P.; Mayer, F.; Galehdari, D.; Weiland, K.; Woodward, R. T., *Small* **2023,** *19* (50), e2304562. DOI 10.1002/smll.202304562.

[2] Blocher, A.; Mayer, F.; Schweng, P.; Tikovits, T. M.; Yousefi, N.; Woodward, R. T., *Mater. Adv.* **2022,** *3* (15), 6335-6342. DOI 10.1039/d2ma00379a.

[3] Thommes, M.; Kaneko, K.; Neimark, A. V.; Olivier, J. P.; Rodriguez-Reinoso, F.; Rouquerol, J.; Sing, K. S. W., *Pure Appl. Chem.* **2015,** *87* (9-10), 1051-1069. DOI 10.1515/pac-2014-1117.

[4] Feitoza, N. C.; Goncalves, T. D.; Mesquita, J. J.; Menegucci, J. S.; Santos, M. K.; Chaker, J. A.; Cunha, R. B.; Medeiros, A. M.; Rubim, J. C.; Sousa, M. H., *J. Hazard. Mater.* **2014,** *264*, 153-60. DOI 10.1016/j.jhazmat.2013.11.022.

[5] Egbosiuba, T. C.; Abdulkareem, A. S., *J. Mater. Res. Technol.* **2021,** *15*, 2848-2872. DOI 10.1016/j.jmrt.2021.09.094.

[6] White, R. L.; White, C. M.; Turgut, H.; Massoud, A.; Tian, Z. R., *J. Taiwan Inst. Chem. Eng.* **2018,** *85*, 18-28. DOI 10.1016/j.jtice.2018.01.036.

[7] Awual, M. R.; Eldesoky, G. E.; Yaita, T.; Naushad, M.; Shiwaku, H.; AlOthman, Z. A.; Suzuki, S., *Chem. Eng. J.* **2015,** *279*, 639-647. DOI 10.1016/j.cej.2015.05.049.

[8] Ren, Y.; Yan, N.; Feng, J.; Ma, J.; Wen, Q.; Li, N.; Dong, Q., *Mater. Chem. Phys.* **2012,** *136* (2-3), 538-544. DOI 10.1016/j.matchemphys.2012.07.023.

[9] Shahzad, A.; Rasool, K.; Miran, W.; Nawaz, M.; Jang, J.; Mahmoud, K. A.; Lee, D. S., *ACS Sustainable Chem. Eng.* **2017,** *5* (12), 11481-11488. DOI 10.1021/acssuschemeng.7b02695.

[10] Alsabagh, A. M.; Fathy, M.; Morsi, R. E., *RSC Adv.* **2015,** *5* (69), 55774-55783. DOI 10.1039/c5ra07477k.

[11] Gong, J.-L.; Wang, X.-Y.; Zeng, G.-M.; Chen, L.; Deng, J.-H.; Zhang, X.-R.; Niu, Q.-Y., *Chem. Eng. J.* **2012,** *185-186*, 100-107. DOI 10.1016/j.cej.2012.01.050.

[12] Zhang, M., *Chem. Eng. J.* **2011,** *172* (1), 361-368. DOI 10.1016/j.cej.2011.06.017.

[13] Barati, A.; Asgari, M.; Miri, T.; Eskandari, Z., *Environ. Sci. Pollut. Res.* **2013,** *20* (9), 6242-55. DOI 10.1007/s11356-013-1672-3.

[14] Chen, H. W.; Chang, S. H., *Environ. Technol.* **2022,** *43* (6), 805-814. DOI 10.1080/09593330.2020.1805027.
